# Post-analysis methods for lactate threshold depend on training intensity and aerobic capacity in runners. An experimental laboratory study: Métodos de pós-análise do limiar do lactato dependem da intensidade de treinamento e da capacidade aeróbica dos corredores. Um estudo laboratorial experimental

**DOI:** 10.1590/1516-3180.2014.8921512

**Published:** 2015-11-13

**Authors:** Tiago Lazzaretti Fernandes, Rômulo dos Santos Sobreira Nunes, Cesar Cavinato Cal Abad, Andrea Clemente Baptista Silva, Larissa Silva Souza, Paulo Roberto Santos Silva, Cyro Albuquerque, Maria Cláudia Irigoyen, Arnaldo José Hernandez

**Affiliations:** I MD, MSc. Doctoral Student and Attending Physician, Sports Medicine Group, FIFA Medical Centre of Excellence, Faculdade de Medicina da Universidade de São Paulo (FMUSP), and Instituto de Ortopedia e Traumatologia (IOT), Hospital das Clínicas (HC), São Paulo, Brazil.; II Undergraduate Student, Faculdade de Medicina da Universidade de São Paulo (FMUSP), São Paulo, Brazil.; III MSc, PhD. Heart Institute, Faculdade de Medicina da Universidade de São Paulo (FMUSP), and Instituto do Coração (InCor), São Paulo, Brazil.; IV MD. Sports Medicine Group, Faculdade de Medicina da Universidade de São Paulo (FMUSP), and Instituto de Ortopedia e Traumatologia (IOT), Hospital das Clínicas (HC), São Paulo, Brazil.; V PhD. Sports Medicine Group, FIFA Medical Centre of Excellence, Faculdade de Medicina da Universidade de São Paulo (FMUSP), and Instituto de Ortopedia e Traumatologia (IOT), Hospital das Clínicas (HC), São Paulo, Brazil.; VI MSC, PhD. Assistant Professor, Department of Mechanical Engineering, Centro Universitário da FEI, São Bernando do Campo, Brazil.; VII MD, PhD. Professor, Faculdade de Medicina da Universidade de São Paulo (FMUSP), and Instituto do Coração (InCor), São Paulo, Brazil.; VIII PhD. Assistant Professor, Director of Sports Medicine Group, FIFA Medical Centre of Excellence, Faculdade de Medicina da Universidade de São Paulo (FMUSP), and Instituto de Ortopedia e Traumatologia (IOT), Hospital das Clínicas (HC), São Paulo, Brazil.

**Keywords:** Lactic acid, Physical endurance, Anaerobic threshold, Oxygen consumption, Exercise test, Sports medicine

## Abstract

**CONTEXT AND OBJECTIVE:**

This study aimed to evaluate different mathematical post-analysis methods of determining lactate threshold in highly and lowly trained endurance runners.

**DESIGN AND SETTING:**

Experimental laboratory study, in a tertiary-level public university hospital.

**METHOD:**

Twenty-seven male endurance runners were divided into two training load groups: lowly trained (frequency < 4 times per week, < 6 consecutive months, training velocity ≥ 5.0 min/km) and highly trained (frequency ≥ 4 times per week, ≥ 6 consecutive months, training velocity < 5.0 min/km). The subjects performed an incremental treadmill protocol, with 1 km/h increases at each subsequent 4-minute stage. ­Fingerprint ­blood-lactate analysis was performed at the end of each stage. The lactate threshold (i.e. the running velocity at which blood lactate levels began to exponentially increase) was measured using three different methods: increase in blood lactate of 1 mmol/l at stages (DT1), absolute 4 mmol/l blood lactate concentration (4 mmol), and the semi-log method (semi-log). ANOVA was used to compare different lactate threshold methods and training groups.

**RESULTS:**

Highly trained athletes showed significantly greater lactate thresholds than lowly trained runners, regardless of the calculation method used. When all the subject data were combined, DT1 and semi-log were not different, while 4 mmol was significantly lower than the other two methods. These same trends were observed when comparing lactate threshold methods in the lowly trained group. However, 4 mmol was only significantly lower than DT1 in the highly trained group.

**CONCLUSION:**

The 4 mmol protocol did not show lactate threshold measurements comparable with DT1 and semi-log protocols among lowly trained athletes.

## RESUMO

**CONTEXTO E OBJETIVO:**

O objetivo do presente estudo é avaliar modelos matemáticos de pós-análise do limiar de lactato em grupos de corredores de longa distância muito ou pouco treinados.

**TIPO DE ESTUDO E LOCAL:**

Estudo laboratorial experimental. Hospital Público Universitário Terciário.

**MÉTODO:**

Vinte e sete corredores homens foram divididos em: pouco treinados (frequência < 4 vezes por semana, < 6 meses, velocidade ≥ 5,0 minutos/km) e muito treinados (frequência ≥ 4 vezes por semana, ≥ 6 meses, velocidade < 5,0 minutos/km). Os participantes foram submetidos a protocolo de esteira escalonado (1% inclinação) = 1 km/h por fase (4 minutos). Ao fim de cada estágio, análise da “impressão digital” metabolômica foi realizada. O limiar do lactato (i.e. velocidade em que o lactato sanguíneo aumenta exponencialmente) foi medido utilizando-se três métodos: aumento de 1 mmol/l da concentração, concentração absoluta de 4 mmol e método semi-log. ANOVA foi utilizada para comparar os diferentes limiares de lactato e grupos.

**RESULTADO:**

Atletas muito treinados apresentaram limiares de lactato maiores que os corredores pouco treinados, independentemente do método de cálculo utilizado. Comparando todos os corredores juntos, as análises de aumento de 1 mmol/l e semi-log não foram diferentes, enquanto a concentração absoluta de 4 mmol/l foi significativamente mais baixa que as dos dois outros métodos. Essas mesmas tendências foram observadas ao se compararem os métodos de limiar de lactato no grupo menos treinado. Entretanto, a análise absoluta de 4 mmol/l foi menor do que a do aumento de 1 mmol/l no grupo muito treinado.

**CONCLUSÃO:**

O método concentração absoluta de 4 mmol não mostrou mensurações comparáveis de limiar do lactato quando comparado com os protocolos aumento de 1 mmol/l e semi-log nos atletas pouco treinados.

## INTRODUCTION

Blood lactate evaluation commonly complements endurance training regimens.^1^^,^^2^ It has been recommended as an efficient method for evaluating training intensity and recovery, and for improving the performance of endurance athletes.^3^^,^^4^^,^^5^^,^^6^ During incremental exercise, the lactate threshold (LT) is defined as the abrupt transition from slow increases to rapid exponential increases in blood lactate levels.^7^

The evaluation of lactate threshold in athletes has evolved, from the 4 mmol universal lactate threshold, to the more individualized Onset of Blood Lactate Accumulation, and to the current Maximal Lactate Steady State standard. This progression has been due to better understanding of the physiological processes of lactate production and clearance, and the role of lactate during prolonged and submaximal exercise.^5^^,^^8^^,^^9^^,^^10^^,^^11^^,^^12^

However, most published studies on lactate threshold have compared homogeneous groups of athletes with similar aerobic capacity, or have made regression analyses on these ­data.^13^^,^^14^^,^^15^^,^^16^ Comparisons between different methods on lactate threshold acquisition also remain controversial in the literature.^17^^,^^18^^,^^19^ To our knowledge, there is no comparative study evaluating lactate threshold methods in both lowly and highly trained endurance athletes.

## OBJECTIVE

The purpose of this study was to evaluate different lactate threshold methods, and determine which methods are most reliable for athletes with different physical conditioning and training programs.

## METHODS

This was an experimental laboratory study performed within the Sports Medicine Group of Faculdade de Medicina da Universidade de São Paulo. Twenty-seven male endurance runners were recruited for this study from university campus running clubs. For the primary outcome (post-analysis method for the lactate threshold in the same group), the sample size was calculated after a five-athlete pilot study, taking P < 0.05 and power = 80%. The sample size was estimated as 10 individuals per group. We added a minimum of 20% more subjects to account for potential data loss.

The subjects were divided into two distinct groups based on the responses to a questionnaire: 15 highly trained runners (minimum of 4 training runs per week for 6 consecutive months, and a long-distance training pace less than or equal to 5.0 min/km) and 12 lowly trained runners (long-distance training pace greater than 5.0 min/km, with a maximum of 3 runs per week and a maximum of 6 consecutive training months). The exclusion criteria were previous cardiorespiratory disease and musculoskeletal running-related injuries. No athlete was currently taking any medications.

Oxygen consumption (VO_2_) was measured continuously and monitored by means of a breath-by-breath gas analyzer on a treadmill (h/p/cosmos, Pulsar, Germany) using a metabolic analyzer (CPX/D Med Graphics, St. Paul, USA)

The mean physiological characteristics of the highly trained group were: age 33.7 ± 10.3 years; training velocity: 4.0 ± 0.6 min/km; resting heart rate 68.7 ± 14.7 bpm; and VO_2max_: 52.4 ± 5.3 ml/kg/min. Characteristics of the lowly trained group were: age 37.2 ± 9.3 years; training velocity: 5.3 ± 0.9 min/km; rest heart rate 79.3 ± 15.2 bpm; and VO_2max_: 43.4 ± 5.7ml/kg/min.

The Institutional Review Board approved this research and informed consent was obtained from each subject prior to participation. This research followed the Helsinki Declaration principles.^20^

### Lactate protocol

A washout period of 24 hours with no physical activity was requested for all participants prior to the experiment. The subjects then performed an incremental treadmill test to directly measure their lactate threshold. All subjects did the test at the same location, with the same equipment, and under similar thermal ­conditions (temperature 21-26°C, humidity 33-66%, barometric pressure 688 mmHg). Throughout the protocol, treadmill elevation was kept constant at a 1% grade to duplicate the energy cost of over-ground running.^21^

The subjects first performed a 3-minute warm-up run at 30% of their long-distance training velocity. At the beginning of the incremental test, the treadmill velocity was set at 70% of the estimated long distance training velocity, depending on the running ability of each participant (it is known that performance in competition is an appropriate criterion for valid laboratory tests).^3^^,^^22^

Heart rate and Borg scale were recorded each minute. Stage length was set at 4 minutes,^21^ with running velocity increases of 1 km/h per stage until volitional exhaustion was reached (as measured from the Borg scale). Fingerprint whole-blood samples were taken between the points of 3.5 and 4 minutes in each stage and were immediately analyzed in an automated blood-lactate analyzer (Accutrend Lactate, Typ3012522) without treadmill protocol interruption. Blood samples were collected for two additional stages following exponential inflection of the lactate point.

### Calculating lactate threshold

The basis for determining the lactate threshold is that there is an inflection point at a given workload (i.e. running velocity) where blood lactate exponentially increases with a corresponding increase in workload.^17^^,^^18^^,^^21^^,^^23^^,^^24^ It is used to deﬁne the ­highest work rate or O_2_ uptake (oxygen consumption) at which athletes can maintain their efforts over a speciﬁed time frame.^25^ An individual blood-lactate profile was created for each subject by plotting running velocity (km/h) at each stage of the test (x-axis) versus blood-lactate concentration attained at each stage (y-axis).^10^^,^^21^^,^^24^^,^^26^^,^^27^

**Figure 1.**
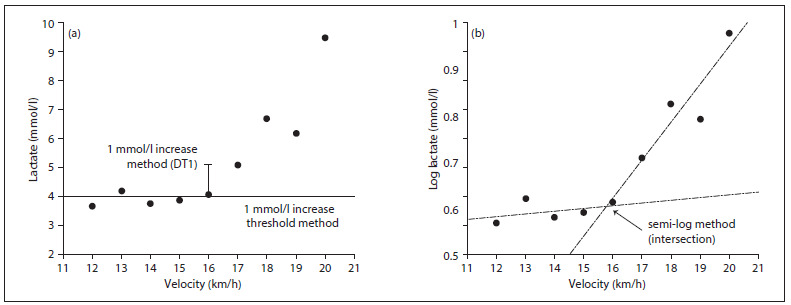
Example of the three post-analysis methods for lactate threshold applied to one subject: (a) the velocity before the increase of 1 mmol/l blood lactate (DT1); the velocity at which the blood lactate exceeds the value of 4 mmol/l (4 mmol); and (b) the velocity at the intersection of two interpolated lines on the semi-logarithmic scale (semi-log).

Three methods commonly cited in the literature were used to define the inflection point (Figure 1):

1. Increase of 1 mmol/l blood lactate (DT1): the work rate that just precedes a rise in blood lactate concentration of > 1 mmol/l between two stages estimates the lactate threshold.^10^^,^^21^^,^^26^^,^^27^

2. Absolute value of 4 mmol/l blood lactate (4 mmol): workload when the concentration of lactate in the blood reaches 4 mmol/l.^10^^,^^21^^,^^26^^,^^27^

3. Semi-log method (semi-log): based on a logarithmic scale (blood lactate) in which the exponential blood lactate curve is divided into two linear segments that cross each other; the point of intersection is the lactate threshold.^17^^,^^18^

### Statistical analysis

The normality curve was addressed by means of histograms and it was decided to use parametric tests. Baseline characteristics were analyzed first to demonstrate homogeneity. The threshold values of each method were compared with repeated-measurement analyses of variance (ANOVA). When a significant difference was attained, Tukey’s post-hoc test was performed. Statistical significance was denoted as P < 0.05 (STATA-9 for Windows).

## RESULTS

Before analyzing the relationship between lactate threshold and velocity, the associations between lactate threshold and baseline characteristics such as age, heart rate, VO_2max_ and training regularity were assessed. This analysis showed that neither demographic nor baseline characteristics could explain associations with lactate threshold, except, logically, for the dependent variables of training regularity between groups and VO_2max_.

**Figure 2.**
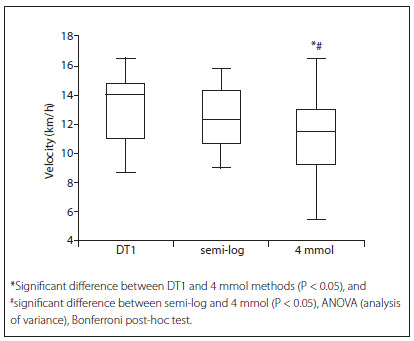
Box plot of the velocity of the lactate threshold of all subjects obtained using the DT1, semi-log and 4 mmol post-analysis methods.

As expected, the lactate thresholds of the highly trained group were obtained at higher velocity stages than those of the lowly trained group in all tested methods. When considering all subjects (both the highly trained and the lowly trained groups), comparison of lactate threshold methods showed significant differences between the DT1 and 4 mmol methods, and between the semi-log and 4 mmol methods. There was no statistical difference between DT1 and semi-log (Figure 2).

**Figure 3.**
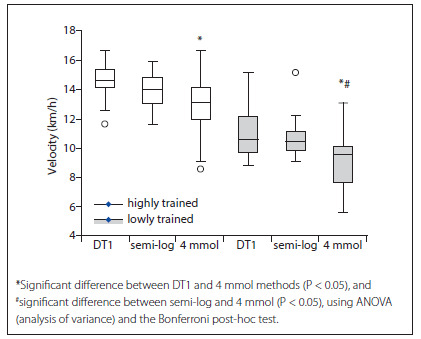
Box plot of the velocities at the lactate threshold obtained using the DT1, semi-log and 4 mmol post-analysis methods in each group (highly trained and lowly trained).

When the groups were compared separately (highly trained and lowly trained), the 4 mmol measurement was found to be significantly lower than the DT1 and semi-log measurements in the lowly trained group. The DT1 and semi-log measurements were not statistically different in this group (Figure 3). In the highly trained group, a significant difference was only found between the DT1 and the 4 mmol methods.

## DISCUSSION

The most important finding of this study was the differences in lactate threshold measurement methods between highly and lowly trained endurance runners. The method with fixed blood lactate of 4 mmol/l underestimated the lactate threshold in the lowly trained group.

Sargent et al.^28^ identified differences in lactate threshold between different groups of subjects, such as men versus women. On the other hand, Smekal et al.^29^ reported that blood lactate concentration at the maximal lactate steady state was independent of both endurance capacity and sex. Other authors have showed comparisons between trained and untrained individuals through using cardiorespiratory tests.^30^^,^^31^ One notable characteristic of our study is that we only used male subjects and made comparisons between controlled training levels (high and low) instead of between trained and sedentary subjects.

One explanation for the different values of measurement methods is that error is introduced when the curves do not follow the mathematical physiological functions.^19^

Subjects with different performance levels often have different mechanical running responses and consequently different metabolic demands.^32^ Our study also agreed with the literature regarding higher lactate threshold values in trained individuals. Kumagai et al.^33^ showed that aerobic training increased the lactate threshold, with a concomitant improvement in both endurance and middle-distance performance.

Individuals with greater endurance capacity have faster oxygen kinetics.^34^ The higher values of lactate thresholds in the highly trained subjects may reflect more efficient peripheral and central exchange during exercise.^34^ During low-intensity exercise, blood lactate formation and removal depends on the intracellular/tissue balance among the glycolytic (cytosol) and oxidative (mitochondria) processes.^35^ It seems that in trained individuals, these variables are more predictable and have controlled behavior.

Joyner et al.^36^ suggested that running performance could be explained by VO_2_max, running economy and fractional utilization of VO_2_max. Moreover, they suggested that the lactate threshold ­integrates all three of these variables and is the best physiological predictor of distance running performance^1^, given that it is detectable in both trained and untrained individuals.^10^

It is known that the average value for the lactate threshold in normal subjects is 3.7 mmol and that serum blood lactate at the lactate threshold is not equal for all individuals (range: 1.5 to 7.5 mmol) and also changes in a single individual.^37^ Although the 4 mmol lactate protocol is an easy method for estimating lactate threshold, the fixed value of 4.0 mmol does not take these physiological conditions into consideration and may underestimate lactate threshold, as shown in this study.^38^

The clinical relevance of this study relates to the populations tested. Most people are not competitive endurance athletes, yet still need predictions of aerobic threshold and exercise prescriptions for health issues. Our results suggest that lowly trained subjects would benefit from semi-log or DT1 lactate threshold methods in clinical practice.

The main limitation of this study relates to the treadmill protocol, such as the stage duration and initial running velocity. Due to the large variation of treadmill protocols in the ­lactate threshold literature, direct comparisons of our results with previous studies may not be appropriate. Despite training group characteristics that were very specific (frequency, intensity and duration of training), we believe that they represent objective inclusion criteria and, because of that, the results may be reproducible. Future studies should examine lactate threshold methods and cardiorespiratory performance in both highly trained and lowly trained groups. We suggest that these groups should be stratified according to training frequency, intensity and duration.

## CONCLUSION

The 4 mmol protocol did not show lactate threshold measurements comparable with with DT1 and semi-log protocols among lowly trained athletes.
